# Water-Soluble O-, S- and Se-Functionalized Cyclic Acetyl-triaza-phosphines. Synthesis, Characterization and Application in Catalytic Azide-alkyne Cycloaddition

**DOI:** 10.3390/molecules25225479

**Published:** 2020-11-23

**Authors:** Abdallah G. Mahmoud, Piotr Smoleński, M. Fátima C. Guedes da Silva, Armando J. L. Pombeiro

**Affiliations:** 1 Department of Chemistry, Faculty of Science, Helwan University, Ain Helwan, 11795 Cairo, Egypt; 2 Centro de Química Estrutural, Instituto Superior Técnico, Universidade de Lisboa Av. Rovisco Pais, 1049-001 Lisboa, Portugal; fatima.guedes@tecnico.ulisboa.pt (M.F.C.G.d.S.); pombeiro@tecnico.ulisboa.pt (A.J.L.P.); 3 Faculty of Chemistry, University of Wrocław, Ul. F. Joliot-Curie 14, 50-383 Wrocław, Poland; piotr.smolenski@chem.uni.wroc.pl

**Keywords:** P ligands, water-soluble ligands, homogeneous catalysis, click chemistry, DAPTA, CuAAC

## Abstract

The 3,7-diacetyl-1,3,7-triaza-5-phosphabicyclo[3.3.1]nonane (DAPTA) derivatives, viz. the already reported 3,7-diacetyl-1,3,7-triaza-5-phosphabicyclo[3.3.1]nonane 5-oxide (DAPTA=O, **1**), the novel 3,7-diacetyl-1,3,7-triaza-5-phosphabicyclo[3.3.1]nonane-5-sulfide (DAPTA=S, **2**), and 3,7-diacetyl-1,3,7-triaza-5-phosphabicyclo[3.3.1]nonane-5-selenide (DAPTA=Se, **3**), have been synthesized under mild conditions. They are soluble in water and most common organic solvents and have been characterized using ^1^H and ^31^P NMR spectroscopy and, for **2** and **3**, also by single crystal X-ray diffraction. The effect of O, S, or Se at the phosphorus atom on the structural features of the compounds has been investigated, also through the analyses of Hirshfeld surfaces. The presence of **1**–**3** enhances the activity of copper for the catalytic azide-alkyne cycloaddition reaction in an aqueous medium. The combination of cheaply available copper (II) acetate and compound **1** has been used as a catalyst for the one-pot and 1,4-regioselective procedure to obtain 1,2,3-triazoles with high yields and according to ‘click rules’.

## 1. Introduction

Decreasing the environmental impact of noxious organic solvents on industrial-scale applications constitutes one of the most imperative research topics in response to the growing environmental and sustainability concerns. Solvent-free chemical processes might seem to be an attractive alternative, but solvents play a tremendous role in controlling the equilibrium and rates of reactions. Alternative “green solvents” have been developed, such as bioethanol, glycerol, polyethylene glycol, 2-methyltetrahydrofuran, ethyl lactate, dihydrolevoglucosenone (cyrene), supercritical fluids, and ionic liquids [1,2]. Water is the best choice among them due to its environmental compatibility, high abundance, nonflammability and nontoxicity. Besides the environmental impact, the rapid development of aqueous-phase catalysis is attributed to several advantages including: (1) facile and efficient separation of the water-soluble catalyst from the organic reactants and products by simple phase separation, (2) easy recyclability of the catalyst using the aqueous catalytic phase, and (3) mild reaction conditions [3,4,5].

**Figure 1:**
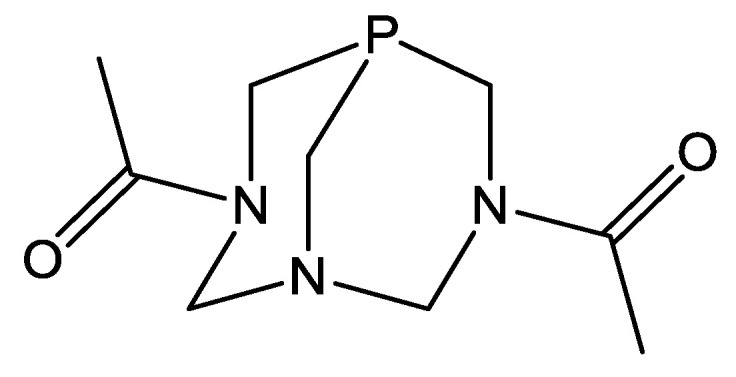
3,7-diacetyl-1,3,7-triaza-5-phosphabicyclo[3.3.1]nonane (DAPTA).

Considerable efforts have been directed towards developing metal complexes with hydrosoluble ligands, in particular phosphines [3]. Moreover, 1,3,5-triaza-7-phosphaadamantane (PTA) and derivatives are recognized as one of the most useful types of water soluble phosphines for coordination chemistry [6,7]. The di-*N*-acylated derivative of PTA, 3,7-diacetyl-1,3,7-triaza-5-phosphabicyclo[3.3.1]nonane (DAPTA; Figure 1), has acquired the most significant attention when compared to the other PTA analogues due to its incomparable high solubility in water (7.4 M) [8]. Several transition metal complexes bearing DAPTA ligands have been synthesized and used in aqueous-phase catalysis [9].

**Scheme 1:**
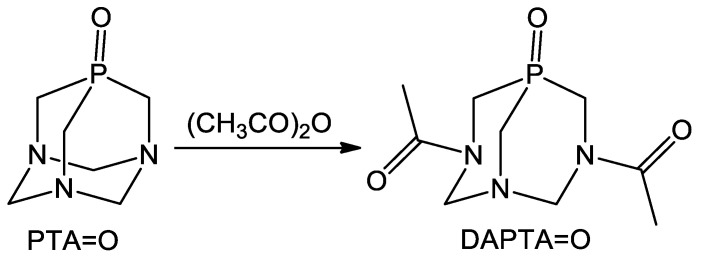
Synthesis of DAPTA=O by acylation of PTA=O.

The only P-substituted derivative of DAPTA that has been obtained so far is the oxidized analogue, 3,7-diacetyl-1,3,7-triaza-5-phosphabicyclo[3.3.1]nonane 5-oxide (DAPTA=O). It was prepared by Siele in 1977, [10] by the acylation of 1,3,5-triaza-7-phosphaadamantane-7-oxide (PTA=O) with acetic anhydride (Scheme 1). DAPTA=O is a highly water-soluble and stable compound. However, its coordination properties and applications in catalysis remained unexplored until our group has recently reported the copper {[Cu(μ-CH_3_COO)_2_(κ*O-*DAPTA=O)]_2_} and sodium {[Na(μ-1κ*OOʹ*;2κ*O-*DAPTA=O)(MeOH)]_2_(BPh_4_)_2_} complexes, with the former being catalytically active for alcohol oxidation and nitro-aldol condensation in aqueous media [11].

No other studies of this P-functionalization of DAPTA have been reported [9]. Due to the increasing demands for a larger variety of water-soluble ligands for catalytic applications in aqueous media, we have chosen to investigate the functionalization of DAPTA for this purpose. Herein, we report the synthesis of DAPTA=O (**1**) by direct oxidation of DAPTA, and the new water-soluble compounds, viz. 3,7-diacetyl-1,3,7-triaza-5-phosphabicyclo[3.3.1]nonane-5-sulfide (DAPTA=S; **2**), and 3,7-diacetyl-1,3,7-triaza-5-phosphabicyclo[3.3.1]nonane-5-selenide (DAPTA=Se; **3**). These compounds were characterized in solution by ^1^H and ^31^P-NMR spectroscopy, and in the solid state by single crystal X-ray diffraction (SCXRD) analysis. To pursue our interest in the copper catalyzed azide-alkyne cycloaddition (CuAAC) reaction [12,13,14], compounds **1**–**3** were used for in-situ catalyst generation for CuAAC in aqueous media. For comparative purposes, the study was extended to the well-defined Cu(II) complex [Cu(μ-CH_3_COO)_2_(κ*O-*DAPTA=O)]_2_ (**4**) [11].

## 2. Results and Discussion

### 2.1. Synthesis and Characterization of ***1***–***3***

**Scheme 2:**
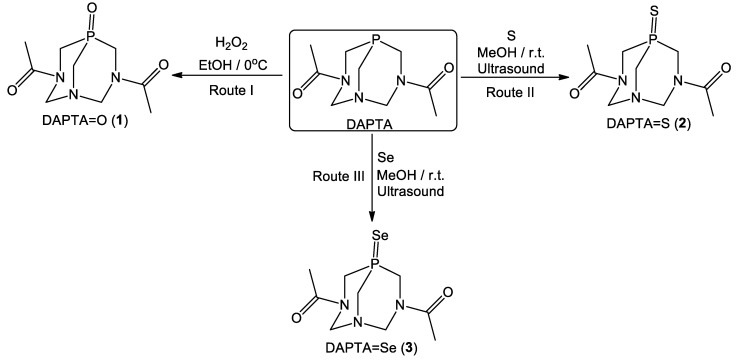
Synthesis of compounds **1**–**3**.

Oxidation of DAPTA with hydrogen peroxide in ethanol leads to the formation of DAPTA=O (**1**) in 79% yield (Scheme 2, route I). Reaction of DAPTA with sulphur or selenium in methanol, under ultrasonic irradiation for 1h and at room temperature afforded DAPTA=S (**2**) or DAPTA=Se (**3**) in good yields, 94 and 64%, respectively (Scheme 2, routes II and III). Compounds **1**–**3** exhibit high solubility in water, dimethyl sulfoxide, methanol, and chloroform, whereas they are not soluble in diethyl ether, benzene, and hexane.

All compounds were characterized by elemental analysis, NMR (^1^H, ^31^P{^1^H}) spectroscopies and ESI-MS, which support the proposed formulations. In addition, their structures were authenticated by SCXRD analysis. The obtained crystal structure of **1** was in agreement with that already reported [8].

**Figure 2:**
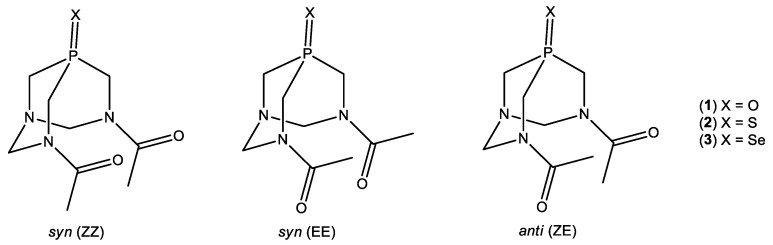
Isomeric forms of compounds **1**–**3**.

**Figure 3:**
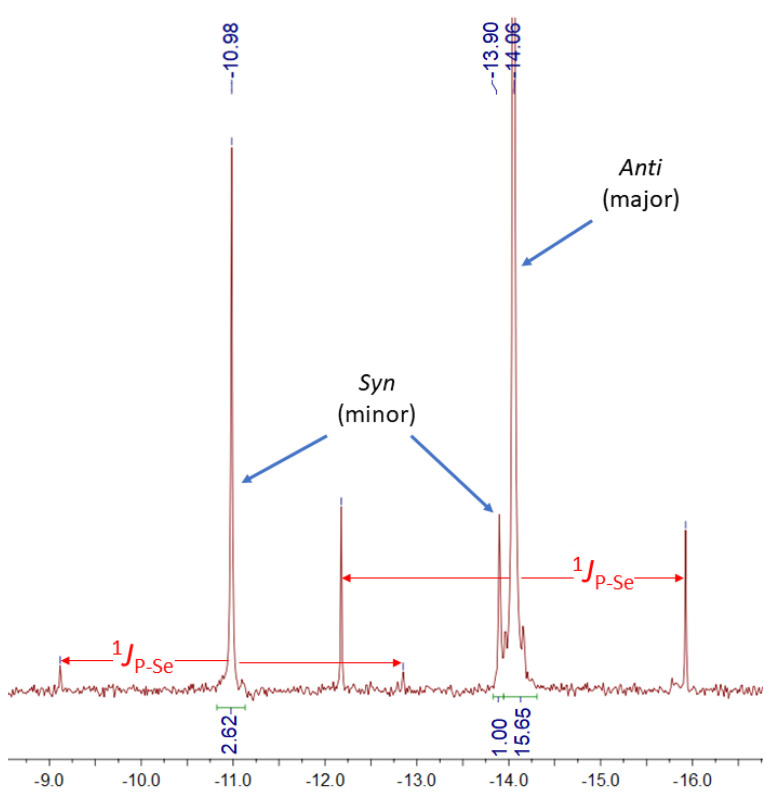
^31^P{^1^H} NMR spectrum of DAPTA=Se (**3**) in DMSO-*d*_6_ (500 MHz).

The ^1^H and ^31^P{^1^H} NMR spectroscopic data were obtained in DMSO-*d*_6_ (see Supplementary Figures S1, S2, S4, S5, and S7). In all cases, the observation of four resonances for the acyl groups in the ^1^H NMR spectra and three independent signals in the ^31^P{^1^H} NMR indicates the presence of the compounds in solution in three rotameric forms (Figure 2). The ^1^H NMR spectrum of **3** (Supplementary Figure S7) shows four singlets for the acyl groups at 2.07, 2.02, 1.97 and 1.94 ppm, with an integration ratio of 1:2.6:7.8:7.8. The resonances at 2.07 and 2.02 ppm are assigned to the two *syn* isomers (minor) of the compound, where each signal represents the equivalent acetyl groups in the same isomer. The pair of more intense signals at 1.97 and 1.94 ppm represent the two non-equivalent acetyl groups of the *anti* isomer (major). The ^31^P{^1^H} NMR spectrum of **3** (Figure 3) shows three singlets, namely those at −10.98 and −13.9 for the *syn* isomers, and that at −14.06 ppm for the *anti* isomer. The intense ratio is 2.6:1:15.6, which is in fair agreement with that observed in the ^1^H NMR spectrum. The calculated first order ^77^Se−^31^P coupling constant (^1^*J*_Se−P_) is of 934 Hz. It is well established that the observation of ^1^*J*_Se−P_ values for phosphine-based compounds allows the assessment of the phosphorus basicity. With DAPTA=Se having a ^1^*J*_Se−P_ value higher than that of its PTA analogue (1,3,5-triaza-7-phosphaadamantane-7-selenide, PTA=Se, 760 Hz), [15] it appears that DAPTA is a weaker σ donor when compared to PTA. Similarly, the ^1^H and ^31^P{^1^H} NMR spectra in DMSO-*d*_6_ for compounds **1** and **2** (Supplementary Figures S1, S2, S4, and S5) are consistent with the presence of the three rotamers in each case, with the *anti* isomer being the major component.

^31^P{^1^H} NMR experiments were also performed in D_2_O (for compounds **1** and **2**; Figures S3 and S6) and in CDCl_3_ (for compound **3**). In all cases, the existence of the three rotamers was confirmed by the presence of three ^31^P resonances.

**Figure 4:**
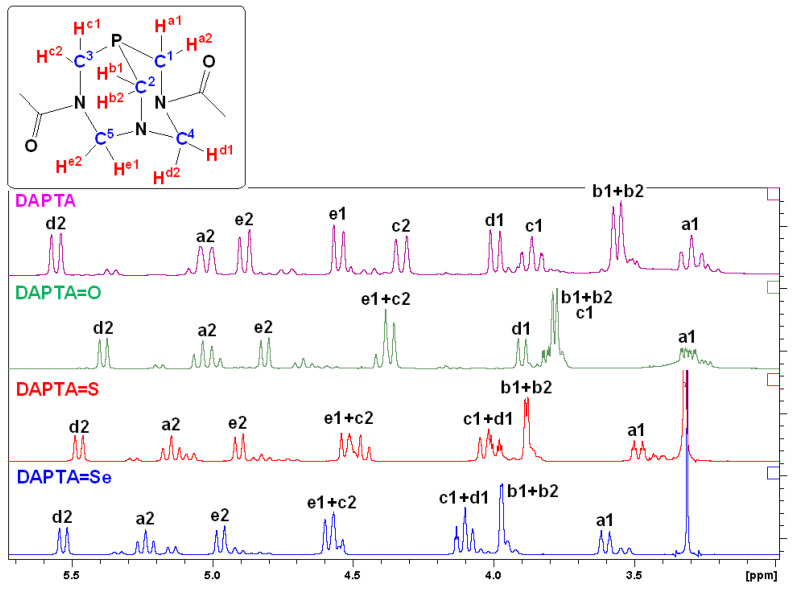
^1^H NMR spectra of DAPTA and compounds **1**–**3** in DMSO-*d*_6_.

In all cases, the ^1^H NMR spectrum shows seven sets of signals arising from the ten aliphatic methylene protons, observed between 5.6 to 3.2 ppm. Their splitting patterns are traceable through the diastereotopic nature of the NC*H*_2_N and PC*H*_2_N moieties. These resonances were assigned based on COSY experiments. Figure 4 shows a comparison of the ^1^H NMR spectra of DAPTA and of compounds **1**–**3** in DMSO-*d_6_* in the 5.7–3.0 ppm region. A comparison of the ^31^P{^1^H} NMR spectra these compounds in the same solvent is depicted in Supplementary Figure S9.

### 2.2. Single Crystal X-ray Diffraction Analysis

**Figure 5:**
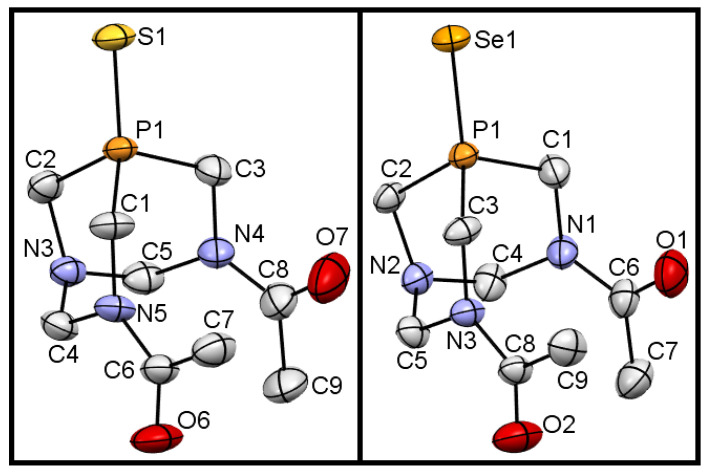
ORTEP diagrams of compounds **2** (left) and **3** (right) with displacement ellipsoids shown at 50% probability level and atom numbering schemes. H-atoms are omitted for clarity.

Single crystals of **2** and **3** were obtained from methanol solution by slow evaporation at room temperature. Compound **2** crystalized in the orthorhombic space group Pbcn, and **3** in the monoclinic space group P21/n. Crystallographic data and structure refinement details are provided in Table S1. Thermal ellipsoid representations are depicted in Figure 5. Table 1 shows the selected bond distances and angles for compounds **2** and **3** and, for comparative reasons, those of DAPTA and DAPTA=O (**1**) obtained from their reported SCXRD structures [8].

The presence of an additional substituent on the phosphorus atom leads to considerable differences in bonding geometry of the crystal structures of DAPTA and its homologs, compounds **1**–**3**. The largest differences in bonding geometry were observed in the bonds that involve the P atom and the adjacent C atoms. The presence of a substituent on the P atom is accompanied by an elongation of the P-C bond distances, an opening of the C-P-C angles and a decreasing of the P-C-N angles (Table 1).

The magnitude of the P–X (X = O, S or Se) bond distances should depend on the electronegativity of X which assumes values of 3.44 (O) >> 2.58 (S) ≈ 2.55 (Se) [16].Consequently, the P-O bond length in **1** is much shorter than those of P-S (in **2**) and P-Se (in **3**). The differences in bonding geometry observed for the DAPTA P^III^ molecule and the oxidized P^V^ species **1**–**3** are consistent with the different oxidation states of phosphorus and electronegativities of X.

**Scheme 3:**
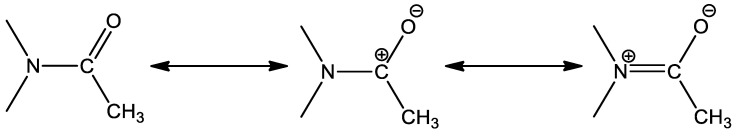
Schiff base N=C bond formation through the π electronic resonance of the amide group.

In all the compounds the acetyl groups adopt the *anti* orientation. The N–C_carbonyl_ bond distances in **2** and **3** (in the 1.350(6)–1.362(6) Å range; Table 1) are shorter than the other N–C bonds (between 1.429(6) and 1.486(6) Å; Table 1), which confirm the double bond character of such Schiff base type moiety (Scheme 3) and justify the solubility in water of the respective compounds. The same was observed for DAPTA and **1** [8].

### 2.3. Hirshfeld Structural Comparison of DAPTA and Derivatives ***1***–***3***

The 3D Hirshfeld analysis and 2D fingerprint plots of DAPTA and compounds **1**–**3** were performed with the CrystalExplorer version 17.5 software [17] and were mapped (Supplementary Figure S10, top) with the *d*_norm_ property where the blue, white and red colours reveal the long, at van der Waals and the short interatomic contacts, respectively. A comparison of the Hirshfeld volumes expectedly shows the consequence of functionalization, with a slight growth upon oxidation of DAPTA (an increase from 262 to 273 Å^3^ was calculated, upon formation of DAPTA=O) and more effective for **2** and **3** (volumes of 303 and 306 Å^3^, in this order) conceivably due to the relatively larger dimensions of S and Se against O. In view of the weak interactions in the crystals, more informative representations could be obtained by means of shape-index measurements in which the convex blue areas identify the donors, and the red concavities the acceptors, as shown in Supplementary Figure S10 (bottom) for the most effective O⋅⋅⋅H contacts.

**Figure 6:**
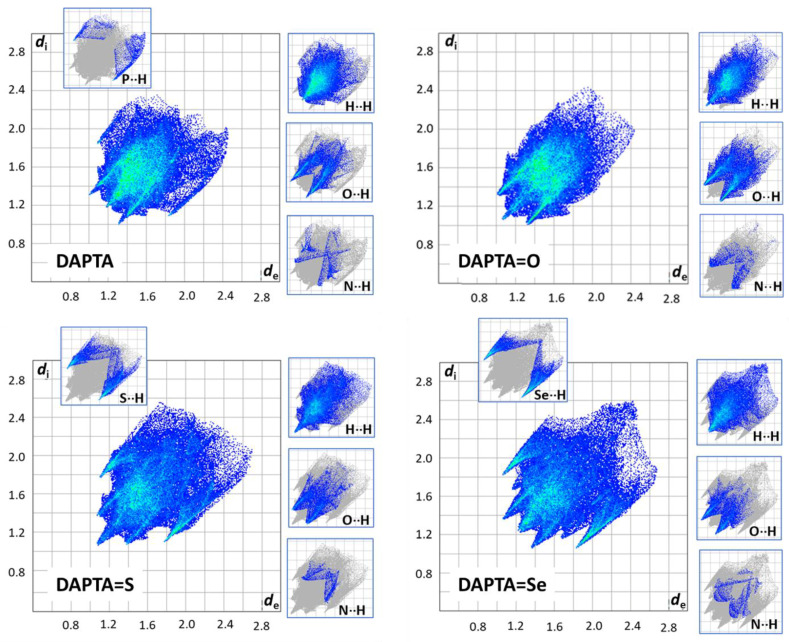
2D fingerprint plots (*d_i_* and *d_e_* values in Å) for DAPTA and P-functionalized derivatives. The offset plots refer to the indicated specific interactions.

**Figure 7:**
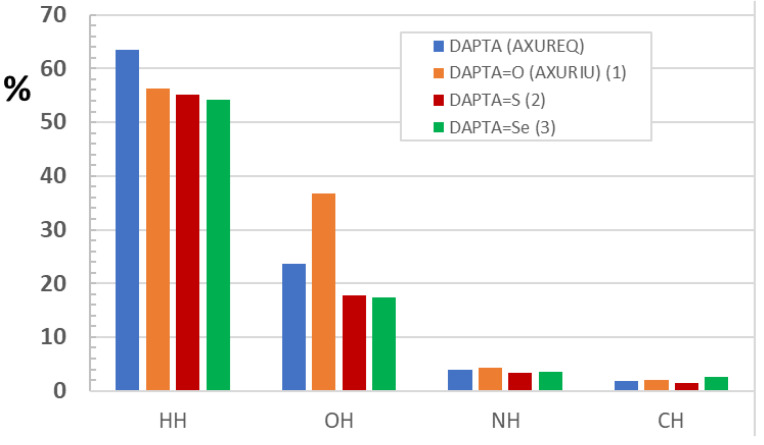
Plot of the percentage contribution of the diverse contacts to the total volume of the surface.

The 2D fingerprint plots [18] in Figure 6 quantitatively describe the nature and type of the intermolecular contacts in the compounds, where *d_i_* and *d_e_* represent the distances from the surface to the nearest atom inside or outside the surface, respectively. The overall 2D plots for DAPTA and **1** are similar (Figure 6), although with a more compact pattern in the latter conceivably due to an increase of the OH contributions to the overall surface (see also Figure 7) and a decrease of the HH ones upon the P-functionalization. The 2D plots for **2** and **3** also have common general features with the higher number of points at larger distances in the latter being due to HH interactions with a short tail at (2.6, 2.4).

The graph in Figure 7 compares the contacts that more extensively contribute to the Hirshfeld volumes in every structure, expectedly showing a much greater influence of the OH contacts in **1** (36.8%) relatively to those in DAPTA (23.6%), compound **2** (17.7%) or **3** (17.3%). The SH and the SeH interactions contribute to 21.0 and 21.4%, respectively.

### 2.4. Catalytic Performances of ***1***–***3*** in CuAAC Reaction

With the aim to develop a highly efficient catalytic system in aqueous medium for CuAAC, cheap and available copper (I) or copper (II) salts were mixed with any of the compounds **1**–**3** and tested for this reaction, first by following well established reaction conditions (see Table 2, legend) which were then adjusted to try to the optimum reaction conditions for this system (Table 2).

For screening the potential catalytic activity of Cu (I) halide salts as a starting point, phenylacetylene and benzyl azide were mixed in water and the system stirred for 24 h. The experiments were performed at room temperature in the presence of 1 mol% of the salt. Under these conditions, the Cu (I) halides were not active (Table 2, entries 1–3), which is not surprising in view of their poor solubility in water. However, the solubility of the copper (I) sources increased upon the addition of compounds **1**–**3** to the reaction media (2 mol%). Under these conditions, a significant catalytic activity was observed (Table 2, entries 4–12), with the mixture of CuI and **1** being the most active one, leading to 62% of the triazole yield (Table 2, entry 4).

Mixtures of different Cu (II) salts with compounds **1**–**3** were also tested as catalysts for the CuAAC reaction (Table 2, entries 13–24). When using CuSO_4_·5H_2_O, the yields were the lowest ones (10–16% range, entries 13–15), whereas Cu(NO_3_)_2_·3H_2_O gave yields from 31 to 39% (entries 16–18), CuBr_2_ led to yields from 48 to 56% (entries 19–21) and Cu(CH_3_COO)_2_·H_2_O improved them to the 55–73% range (entries 22–24). In general, using **1** in the reaction medium with the Cu (I) and Cu (II) salts gives a better catalytic efficiency than using **2** and **3** (Table 2, entries 4–24). Therefore, **1** was used in the following experiments as the optimal choice. A significant drop in the yield of the reaction occurred on decreasing both the catalyst and compound **1** contents to half (i.e., 0.5 mol% of copper acetate and 1 mol% of **1**; Table 2, entry 25), giving 31% conversion after 48 h.

Water-miscible organic co-solvents were used to improve the homogeneity of the reaction system by increasing the solubility of the organic reactants (Table 2, entries 26–30). Using 1:1 water-alcohol mixtures (MeOH, EtOH or ^t^BuOH; entries 26–28) did not improve the yield when compared to that obtained using water as the sole solvent (entry 22). However, a mixture of water and DMF or MeCN significantly increased the conversion to 88% and 97%, respectively (entries 29 and 30). In H_2_O:MeCN (1:1) solvent mixture the completion of the reaction was reached in 8 h when the temperature was raised to 80 °C (Table 2, entry 31).

**Figure 8:**
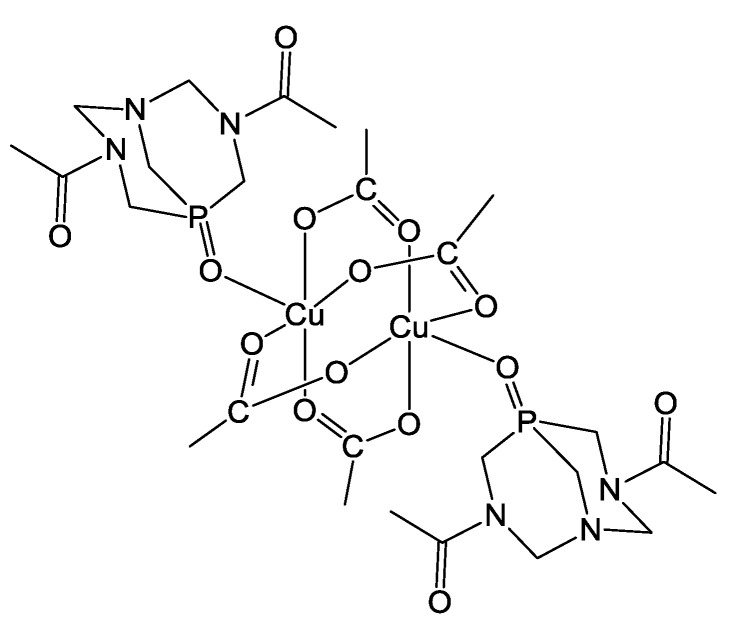
The structure of complex [Cu(μ-CH_3_COO)_2_(κ*O-*DAPTA=O)]_2_ (**4**).

Since the combination of copper acetate and compound **1** gave an optimum catalytic performance for the CuAAC reaction, and the well-defined previously obtained complex [Cu(μ-CH_3_COO)_2_(κ*O-*DAPTA=O)]_2_ [11] (**4**, Figure 8) was included in this study. Using the aforementioned model reaction in H_2_O:MeCN (1:1) solvent mixture, the effects of catalyst loading, reaction time and temperature were investigated (Table 3).

Performing the reaction at room temperature for 24 h, the yield increased from 54% to almost quantitative conversion with increasing the catalyst loading from 0.5 to 5 mol%, while the TON (turnover number = number of moles of product per mol of catalyst) was gradually attenuated (Table 3, entries 1–4). Using 1 mol% of catalyst **4** at room temperature, the reaction yield increased with time to reach 88% after 48 h (Table 3, entries 5–8). The reaction was completed in 6 h with a quantitative conversion when the temperature was raised to 80 °C (Table 3, entry 9). In view of the resemblance of the results (compare entry 31 in Table 2 with entry 9 in Table 3), the active catalytic species in both cases (with **4** or with Cu(CH_3_COO)_2_.H_2_O + **1**) can be the same.

Relying on the optimization studies, the scope of the catalytic system was broadened to include several acetylenes. Various terminal alkynes were reacted with benzyl azide to produce the corresponding 1,4-disubstituted-1,2,3-triazoles (**5**), and the results are summarized in Table 4. The reactions were performed in water:MeCN (1:1) solvent mixture, in the presence of catalyst **4** or the mixture of Cu(CH_3_COO)_2_·H_2_O and compound **1,** under air at 80 °C. The reactions proceeded smoothly to give **5,** usually with similar yields (with **4** or with Cu(CH_3_COO)_2_.H_2_O + **1**) from 81% up to quantitative conversion. The products precipitated from the reaction mixture. After removing the solvent under vacuum, the triazole solids were isolated by filtration, washed, and dried.

Although the position (*meta-* or *para*-) of the substituent group of phenylacetylene seems not to have an influence on the yield of the reaction (compare entries 3–4, with entries 7–8, Table 4), increasing its electron-donating character can decrease that yield (compare entries 7–10 with entries 15–16). However, this is not a general behaviour since the alkyl substituted catalysts lead to higher yields than the fluoro catalyst (compare entries 7–10 and 13–14 with entries 11–12).

In comparison with the copper (I) catalysts with DAPTA=NO(OR)_2_S core ligands for CuAAC reaction, [19] our catalytic system revealed a high catalytic activity to obtain triazoles with quantitative conversions in water/acetonitrile solution after only 6 h, without any added reducing agents or bases. Lower conversions were, however, obtained with water as the sole solvent, but the Cu(I) complexes bearing the iminophosphorane DAPTA derivatives [19] required 10 mol% of 2,6-dimethylpyridine for the reaction to proceed. The Cu (I) complexes [CuX(DAPTA)_3_] and [Cu(μ-X)(DAPTA)_2_]_2_ (X = Br or I) also proved to be of high efficiency with quantitative conversions in aqueous medium under microwave irradiations in 15 min [12]. Despite the high activity of these latter catalysts in terms of very short reaction time, the required copper complex loading was relatively high (5 mol%). The catalyst loading in the present study is low and thus beneficial from the economic point of view, but the higher reaction time is, in this respect, a disadvantage.

Although several N-donor and phosphine ligands were found to form efficient and highly active copper catalysts for CuAAC reactions, [20,21,22] DAPTA=O is notable in view of its high solubility in water, and thus the corresponding complex can be separated easily from the organic product by simple solvent extraction procedures. Unfortunately, complex **4** and the copper (II)/**1** system exhibit poor recyclability as the yield of the triazole product diminished considerably on the following cycles. The obtained yields were 61% and 58% in the second cycle, 15% and 22% in the third cycle for complex **4** and the copper (II)/**1** catalytic systems, respectively.

**Scheme 4:**
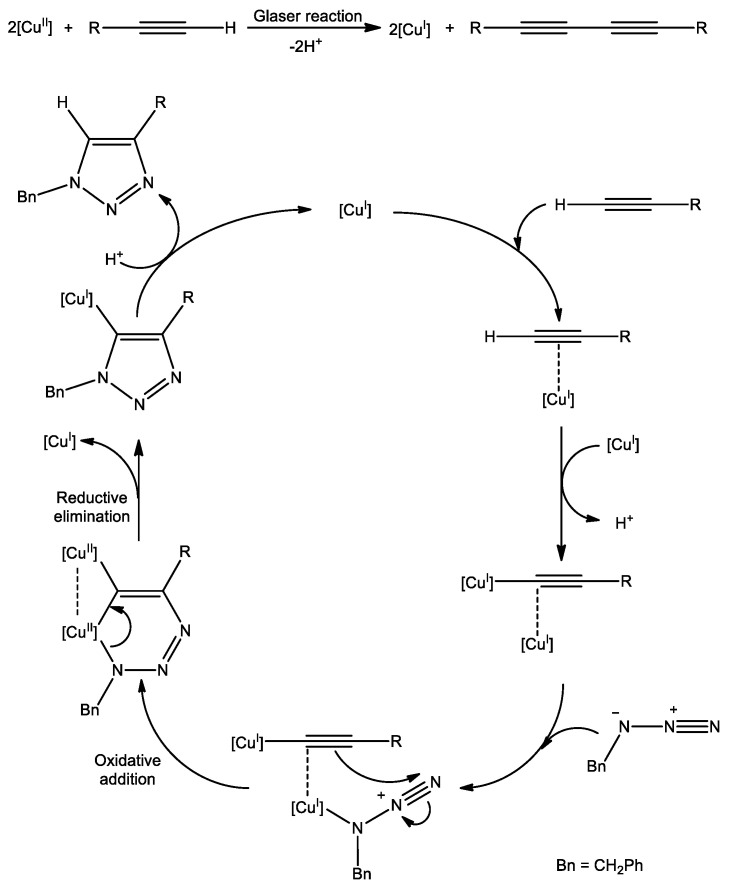
Proposed catalytic cycle for the CuAAC reaction.

A mechanism for the CuAAC reaction has been proposed (Scheme 4) based on fundamental steps established by computational [23,24,25] and experimental methods [26,27,28]. As a starting point, the catalytically active Cu(I) species are generated through an oxidative homocoupling of terminal alkynes (Glaser reaction) [29,30,31,32,33]. The catalytic cycle should start with (1) π-coordination of the alkyne to a Cu (I) species, thus increasing the acidity of the terminal alkyne (pKa drops from ~25 to ~15), and allowing the subsequent (2) formation of a Cu (I) σ-coordinated acetylide, thus generating an intermediate that resembles the known µ-coordination mode of Cu (I) acetylides [34]. (3) A triazolide intermediate is formed upon the coordination of the azide to the π-coordinated Cu (I) center, potentially either through the substituted nitrogen (π-donating) or the terminal one (π-accepting). However, (4) the regioselectivity for the 1,4-isomer was attributed to the π-coordination to Cu (I) of the α-carbon of the acetylide, raising the electron density on the metal centre, directing a nucleophilic attack of the β-carbon at the electrophilic terminal nitrogen and ensuing oxidative coupling. Thus, (5) a six-membered bimetallic cupra-cycle intermediate was proposed, [25] stabilized by a geminal bimetallic coordination. Finally, (6) in an exothermic reductive elimination process, Cu (I) triazolide is formed [25,27], and its protonation releases the 1,4-disubstituted 1,2,3-triazole product.

The catalytic activities of the mixtures of Cu (I) halide salts CuX (I^−^ > Br^−^ > Cl^−^) with **1**–**3** may follow the inverse of the general trend in the spectrochemical series (I^−^ < Br^−^ < Cl^−^), reflecting the π-electron donor ability of the halide, which can promote the oxidation addition step in the catalytic cycle. In the case of mixtures of Cu (II) salts and DAPTA=O (**1**), the highest catalytic activity is observed for the acetate salt. Since the acetate anion is the strongest base in the group, the importance for promoting step (1) of the catalytic cycle is evident. Moreover, acetate readily bridges two copper ions, forming the {Cu_2_(μ-CH_3_COO)_4_} dicopper core (**4**), which promotes the formation of dicopper intermediates involved in the catalytic cycle (Scheme 4).

A different type of effect possibly concerns the extension of O⋅⋅⋅H interactions which decreases in the order DAPTA=O >> DAPTA=S > DAPTA=Se (see above). Such contacts can affect the catalytic cycle, namely assisting in alkyne deprotonation (Scheme 4).

## 3. Materials and Methods

### 3.1. General Procedures

All synthetic procedures were performed in air. Reagents and solvents were obtained from commercial sources. The organic reactants for the cycloaddition reaction (alkynes and benzyl azide) were further purified prior to use by distillation. DAPTA was synthesized using the published procedure [8,10]. The ultrasound irradiation was accomplished with a high-intensity ultrasonic probe SONIC VCX 750 (Sonics & Materials Inc., Newtown, CT, USA) model (20 kHz, 750 W) using a titanium horn. Elemental analyses (C, H, and N) were carried out by the Microanalytical service of Instituto Superior Tecnico. ^1^H and ^31^P NMR spectra were obtained using a Bruker Advance (Bruker, Billerica, MA, USA) 400 and 500 MHz spectrometers at ambient temperature. Chemical shifts δ are quoted in ppm. ^1^H chemical shifts were internally referenced to residual protio-solvent resonance and are reported relative to SiMe_4_. ^31^P chemical shifts were referenced to external 85% phosphoric acid. Assignments of ^1^H signals rely on COSY experiments. Electrospray mass (ESI-MS) spectra were obtained on a Varian 500-MS LC Ion Trap Mass Spectrometer (Agilent Technologies, Santa Clara, CA, USA) equipped with an electrospray ion source. All compounds were observed in the positive mode (capillary voltage = 80–105 V).

### 3.2. Synthesis of Compounds ***1***–***3***

#### 3.2.1. Synthesis 3,7-Diacetyl-1,3,7-Triaza-5-phosphabicyclo[3.3.1]nonane-5-oxide (DAPTA=O, **1**)

To an ethanol solution (20 mL) of DAPTA (500 mg, 2.18 mmol) was added an ethanol solution (20 mL) of hydrogen peroxide (30%, 306.4 µL, 3 mmol) dropwise with stirring over 10 min at 0 °C. The produced colourless solution was further stirred for 30 min at 0 °C. The solvents were removed under vacuum, leaving behind a white solid. The product **1** was recrystallized from ethanol and obtained as a white solid in 78.6% (420 mg) yield.

Elemental analysis calcd (%) for C_9_H_16_N_3_O_3_P: C 44.08, H 6.58, N 17.14; found: C 43.92, H 6.39, N 17.11. ^1^H NMR (500 MHz, DMSO-*d*_6_, δ): 5.39 (d, *J* = 14 Hz, 1H, NC*H*_2_N), 5.04 (dd, *J* = 16 Hz, *J* = 15.5 Hz, 1H, PC*H*_2_N), 4.81 (d, *J* = 14 Hz, 1H, NC*H*_2_N), 4.37–4.35 (m, 2H, NC*H*_2_N + PC*H*_2_N), 3.9 (d, *J* = 13.5 Hz, 1H, NC*H*_2_N), 3.78 (m, 3H, PC*H*_2_N), 3.31 (ddd, ^2^*J*_HP_ = 15.1 Hz, ^2^*J*_HH_ = 8.3 Hz, ^4^*J*_HH_ = 2.5 Hz, 1H, PC*H*_2_N), 2.03 (s, C(O)C*H*_3_, minor-*syn*), 2.00 (s, C(O)C*H*_3_, minor-*syn*), 1.94 and 1.93 (s, 6H, C(O)C*H*_3_, major-*anti*). ^31^P{^1^H} NMR (500 MHz, DMSO-*d*_6_, δ): 5.8 (s, *syn*), 3.13 (s, *syn*), 2.94 (s, anti). ^31^P{^1^H} NMR (400 MHz, D_2_O, δ): 12.19 (s, *syn*), 10.18 (s, *anti*), 9.65 (s, syn). ESI (+) MS in H_2_O (*m*/*z* assignment, % intensity): 246 ([{DAPTA=O} + H]^+^, 100), 491 ([2{DAPTA=O} + H]^+^, 27), 754 ([3{DAPTA=O} + H_2_O + H]^+^, 25).

#### 3.2.2. Synthesis 3,7-Diacetyl-1,3,7-triaza-5-phosphabicyclo[3.3.1]nonane-5-sulfide (DAPTA=S, 2) and 3,7-Diacetyl-1,3,7-triaza-5-phosphabicyclo[3.3.1]nonane-5-selenide (DAPTA=Se, 3)

A similar procedure was utilized for the synthesis of **2** and **3**. A mixture of methanol (10 mL), DAPTA (500 mg, 2.18 mmol) and sulfur (flowers of sulfur, 96 mg, 3 mmol for **2**) or selenium (220 mg, 2.8 mmol for **3**) was placed in 100 mL round bottom flask. At room temperature, the mixture was allowed to sonicate for 1 h. The resulting powders were filtered off, washed with ethanol and benzene, and dried under vacuum.

DAPTA=S (Pale yellow solid). Yield = 94% (535 mg). Elemental analysis calcd (%) for C_9_H_16_N_3_O_2_PS: C 41.37, H 6.17, N 16.08, S 12.27; found: C 41.51, H 6.22, N 16.14, S 12.29. ^1^H NMR (500 MHz, DMSO-*d*_6_, δ): 5.47 (d, *J* = 13.5 Hz, 1H, NC*H*_2_N), 5.15 (dd, *J* = 15 Hz, *J* = 14.5 Hz, 1H, PC*H*_2_N), 4.91 (d, *J* = 14 Hz, 1H, NC*H*_2_N), 4.54–4.47 (m, 2H, NC*H*_2_N + PC*H*_2_N), 4.05–3.98 (m, 2H, NC*H*_2_N + PC*H*_2_N), 3.88 (d, 2H, *J* = 4.5 Hz, PC*H*_2_N), 3.49 (dt, ^2^*J*_HP_ = 15 Hz, ^2^*J*_HH_ = 15 Hz, ^4^*J*_HH_ = 3.5 Hz, 1H, PC*H*_2_N), 2.06 (s, C(O)C*H*_3_, minor-*syn*), 2.02 (s, C(O)C*H*_3_, minor-*syn*), 1.96 and 1.95 (s, 6H, C(O)C*H*_3_, major-*anti*). ^31^P{^1^H} NMR (500 MHz, DMSO-*d*_6_, δ): 2.94 (s, *syn*), −0.35 (s, *syn*), −0.44 (s, anti). ^31^P{^1^H} NMR (400 MHz, D_2_O, *δ*): 3.43 (s, *syn*), 1.46 (s, *anti*), −0.69 (s, syn). ESI (+) MS in H_2_O (*m*/*z* assignment, % intensity): 262 ([{DAPTA=S} + H]^+^, 100), 523 ([2{DAPTA=S} + H]^+^, >5).

DAPTA=Se (Grey solid). Yield = 64% (430 mg). Elemental analysis calcd (%) for C_9_H_16_N_3_O_2_PSe: C 35.08, H 5.23, N 13.64; found: C 34.94, H 5.17, N 13.51. ^1^H NMR (500 MHz, DMSO-*d*_6_, δ): 5.53 (d, *J* = 13.5 Hz, 1H, NC*H*_2_N), 5.24 (dd, *J* = 14.5 Hz, *J* = 14 Hz, 1H, PC*H*_2_N), 4.97 (d, *J* = 14.5 Hz, 1H, NC*H*_2_N), 4.6–4.57 (m, 2H, NC*H*_2_N + PC*H*_2_N), 4.14–4.08 (m, 2H, NC*H*_2_N + PC*H*_2_N), 3.97 (d, 2H, *J* = 2.5 Hz, PC*H*_2_N), 3.6 (dt, ^2^*J*_HP_ = 15 Hz, ^2^*J*_HH_ = 15 Hz, ^4^*J*_HH_ = 2.5 Hz, 1H, PC*H*_2_N), 2.07 (s, C(O)C*H*_3_, minor-*syn*), 2.02 (s, C(O)C*H*_3_, minor-*syn*), 1.97 and 1.95 (s, 6H, C(O)C*H*_3_, major-*anti*). ^31^P{^1^H} NMR (500 MHz, DMSO-*d*_6_, δ): −10.98 (s, *syn*), −13.9 (s, *syn*), −14.06 (s, anti). ^31^P{^1^H} NMR (400 MHz, CDCl_3_, δ): −15.34 (s, *syn*), −17.73 (s, *anti*), −20.13 (s, *syn*). ESI(+)MS in H_2_O (*m*/*z* assignment, % intensity): 310 ([{DAPTA=Se} + H]^+^, 100).

### 3.3. X-ray Structure Determination of Compounds

X-ray quality crystals of the compound were immersed in cryo-oil, mounted in a Nylon loop and measured at ambient temperature. Intensity data were collected using a Bruker AXS-KAPPA APEX II PHOTON 100 diffractometer (Bruker, Billerica, MA, USA) with graphite monochromated Mo-Kα (0.71069 Å) radiation. Data were collected using omega scans of 0.5° per frame and full sphere of data were obtained. Cell parameters were retrieved using Bruker SMART [35] software (Bruker, Madison, WI, USA) and refined using Bruker SAINT [35] (Bruker, Madison, WI, USA) on all the observed reflections. Absorption corrections were applied using the SADABS program (University of Gottingen, Göttingen, Germany) [36]. The structures were solved by direct methods using SIR97 package [37] (IUCr) and refined with SHELXL-2014/7 (IUCr, City, State if USA or Canada, Country) [38]. Calculations were performed using the WinGX System-Version 2014.1 (University of Glasgow, Glasgow, UK) [39]. The hydrogen atoms were included in the refinement using the riding-model approximation; U_iso_(H) were defined as 1.2U_eq_ of the parent carbon atoms for methylene residues, and 1.5U_eq_ of the parent carbon atoms for methyl. The unaccounted twinning in **3** was resolved by using TwinRotMat routine of Platon (University of Glasgow, Glasgow, UK) [40]. The generated *.ins and hkl files were used in the refinement. Least square refinements with anisotropic thermal motion parameters for all the non-hydrogen atoms were employed.

Crystallographic data for the structural analysis have been deposited to the Cambridge Crystallographic Data Center (Cambridge, UK) (CCDC 2,040,205 (for **2**) and 2,040,206 (for **3**)).

### 3.4. General Procedure for the Synthesis of 1,2,3-Triazoles

In a 10 mL cylindrical screw capped vial equipped with a small magnetic stirring bar, a mixture of phenylacetylene (1 mmol), azide derivative (1 mmol), catalyst (copper salt {1 mol%} and compounds **1**–**3** {2 mol%}, or complex **4** {0.5–5 mol%}) and 3 mL of solvent was charged. The mixture was stirred under the temperature and for the time periods indicated in Table 2, Table 3 and Table 4. The reaction mixture was then cooled in an ice bath and diluted with 6 mL of water. The obtained triazole product was collected by filtration, washed with water and repeatedly washed with petroleum ether and dried in a vacuum.

The ^1^H NMR spectroscopic data of the triazole products (**5**) are in agreement with those already reported [22,41,42,43,44,45]. Detailed elemental analysis and ^1^H-NMR data are given in the Supplementary Materials, Section 4.

## 4. Conclusions

Hydrosoluble DAPTA=X compounds **1**–**3** (X = O, S or Se, in the same order) were synthesized in high yields under mild conditions. The structural features of the compounds, including the presence of rotameric forms due to the turning around the N-C bond, were studied in solution by ^1^H and ^31^P NMR spectroscopy, and in solid state by SCXRD. Some crystal structure data of DAPTA and **1** were revisited for comparison with the novel compounds **2** and **3**. The effect of hybridization differences of the phosphorus atom and the electronegativity of the substituents on the structural features of the compounds have been studied, with the most significant differences being observed in the bonds that involve the P and the adjacent C atoms. Comparative Hirshfeld studies of DAPTA and compounds **1**–**3** revealed that the OH interactions contribute to ca. 37–17% of the Hirshfeld volume, following the HH contacts that reach 63–54% of that volume.

Compounds **1**–**3** have demonstrated a moderate to high efficiency to enhance the catalytic activity of copper salts for the CuAAC reaction in aqueous medium. The in situ generated catalyst from a combination of copper acetate with ligand **1** is an efficient catalyst for the one-pot CuAAC reaction of terminal alkynes with benzyl azide to selectively obtain the corresponding 1,4-disubstituted-1,2,3-triazoles in yields ranging from 81 to 99% in 8 h at 80 °C. The catalytic activity of the well-defined copper (II) complex bearing the DAPTA=O ligand (**4**) has also been investigated, and under similar conditions, the triazoles were obtained in yield ranging from 86% to 99% in 6 h.

## Figures and Tables

**Table 1:** Selected bond distances (Å) and angles (°).

DAPTA [8]		1 [8]		2		3	
		P-O	1.491(3)	P1-S1	1.9524(7)	P1-Se1	2.096(1)
P-C	1.71(1)	P-C	1.797(3)	P1-C1	1.824(3)	P1-C1	1.821(7)
	1.74(1)		1.817(3)	P1-C2	1.812(2)	P1-C2	1.817(4)
	1.74(2)		1.828(3)	P1-C3	1.819(2)	P1-C3	1.824(5)
				N4-C5	1.471(3)	N1-C4	1.486(6)
				N3-C5	1.448(3)	N2-C4	1.447(8)
				N3-C4	1.452(3)	N2-C5	1.429(6)
				N5-C4	1.469(3)	N3-C5	1.482(5)
				N4-C8	1.356(4)	N1-C6	1.350(6)
				N5-C6	1.359(3)	N3-C8	1.362(6)
				C6-O6	1.227(3)	C6-O1	1.221(6)
				C8-O7	1.212(4)	C8-O2	1.211(6)
				S1-P1-C1	114.61(9)	Se1-P1-C1	114.1(2)
				S1-P1-C2	119.89(8)	Se1-P1-C2	120.8(2)
				S1-P1-C3	112.98(9)	Se1-P1-C3	112.8(2)
C-P-C	93.8(7)	C-P-C	100.4(1)	C1-P1-C2	100.3(1)	C1-P1-C2	100.0(2)
	98.4(7)		100.7(1)	C2-P1-C3	99.9(1)	C2-P1-C3	100.0(2)
	104.0(7)		106.8(1)	C1-P1-C3	107.3(1)	C1-P1-C3	107.3(2)
				C4-N3-C5	115.8(2)	C4-N1-C6	124.6(4)
				C5-N4-C8	126.1(2)	C4-N2-C5	119.9(4)
				C4-N5-C6	120.0(2)	C5-N3-C8	116.1(4)
P-C-N	115.1(9)	P-C-N	106.2(2)	P1-C2-N3	106.9(2)	P1-C2-N2	105.7(3)
	118(1)		111.8(2)	P1-C1-N5	112.1(2)	P1-C3-N3	112.9(4)
	120.0(9)		114.5(2)	P1-C3-N4	113.5(2)	P1-C1-N1	114.2(4)

**Table 2:**
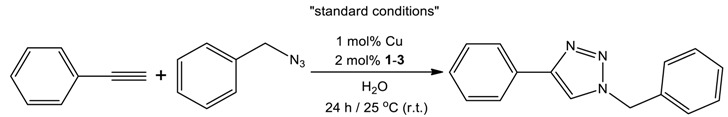
Reaction conditions screening for CuAAC ^a^.

Entry.	Cu Salt	Compound	Change from the “*Standard Conditions*”	Isolated Yield
1	CuI	-	None	0
2	CuBr	-	None	0
3	CuCl	-	None	0
4	CuI	**1**	None	62
5	CuBr	**1**	None	41
6	CuCl	**1**	None	15
7	CuI	**2**	None	56
8	CuBr	**2**	None	36
9	CuCl	**2**	None	7
10	CuI	**3**	None	44
11	CuBr	**3**	None	27
12	CuCl	**3**	None	11
13	CuSO_4_·5H_2_O	**1**	None	16
14	CuSO_4_·5H_2_O	**2**	None	10
15	CuSO_4_·5H_2_O	**3**	None	12
16	Cu(NO_3_)_2_·3H_2_O	**1**	None	39
17	Cu(NO_3_)_2_·3H_2_O	**2**	None	37
18	Cu(NO_3_)_2_·3H_2_O	**3**	None	31
19	CuBr_2_	**1**	None	56
20	CuBr_2_	**2**	None	48
21	CuBr_2_	**3**	None	50
22	Cu(CH_3_COO)_2_·H_2_O	**1**	None	73
23	Cu(CH_3_COO)_2_·H_2_O	**2**	None	58
24	Cu(CH_3_COO)_2_·H_2_O	**3**	None	55
25	Cu(CH_3_COO)_2_·H_2_O	**1**	0.5 mol% of Cu and 1 mol% of **1**, 48 h	31
26	Cu(CH_3_COO)_2_·H_2_O	**1**	H_2_O + MeOH (1:1) solvent mixture	69
27	Cu(CH_3_COO)_2_·H_2_O	**1**	H_2_O + EtOH (1:1) solvent mixture	75
28	Cu(CH_3_COO)_2_·H_2_O	**1**	H_2_O + ^t^BuOH (1:1) solvent mixture	78
29	Cu(CH_3_COO)_2_·H_2_O	**1**	H_2_O + DMF (1:1) solvent mixture	88
30	Cu(CH_3_COO)_2_·H_2_O	**1**	H_2_O + MeCN (1:1) solvent mixture	97
31	Cu(CH_3_COO)_2_·H_2_O	**1**	H_2_O + MeCN (1:1) solvent mixture, 80 °C, 8 h	>99

^a^ Reaction at “standard conditions”: phenylacetylene (1 mmol), benzyl azide (1 mmol) and the catalyst (1 mol% of the Cu salt and 2 mol% of **1**–**3**) were stirred in 3 mL of water at room temperature (25 °C) under air for 24 h.

**Table 3:**
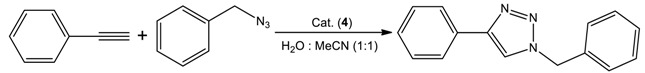
CuAAC reaction using complex **4** as catalyst ^a.^.

Entry	Cat. Load (mol%)	Time (h)	Temp. (°C)	Isolated Yield (%)	TON ^b^
1	0.5	24	25	54	108
2	1	24	25	81	81
3	2	24	25	93	47
4	5	24	25	>99	20
5	1	3	25	34	34
6	1	8	25	49	49
7	1	12	25	57	57
8	1	48	25	88	88
9	1	6	80	>99	100

^a^ Reaction conditions: phenylacetylene (1 mmol), benzyl azide (1 mmol) and 3 mL of water and acetonitrile mixture (1:1). ^b^ Turnover number = number of moles of 1-benzyl-4-phenyl-1*H*-1,2,3-triazole per mol of catalyst.

**Table 4:**
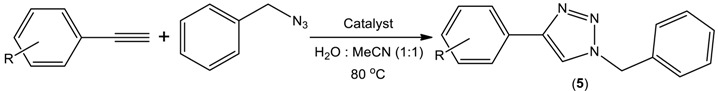
One-pot catalytic synthesis of 1,4-disubstitutred-1,2,3-triazoles ^a.^.

Entry	R	Product	Catalyst	Time (h)	Isolated Yield (%)
1	H	**5**a	Cu(CH_3_COO)_2_·H_2_O (1 mol%) + **1** (2 mol%)	8	>99
2			**4** (1 mol%)	6	>99
3	3-Me	**5**b	Cu(CH_3_COO)_2_·H_2_O (1 mol%) + **1** (2 mol%)	8	93
4			**4** (1 mol%)	6	95
5	3-OMe	**5**c	Cu(CH_3_COO)_2_·H_2_O (1 mol%) + **1** (2 mol%)	8	>99
6			**4** (1 mol%)	6	>99
7	4-Me	**5**d	Cu(CH_3_COO)_2_·H_2_O (1 mol%) + **1** (2 mol%)	8	91
8			**4** (1 mol%)	6	94
9	4-Et	**5**e	Cu(CH_3_COO)_2_·H_2_O (1 mol%) + **1** (2 mol%)	8	88
10			**4** (1 mol%)	6	92
11	4-F	**5**f	Cu(CH_3_COO)_2_·H_2_O (1 mol%) + **1** (2 mol%)	8	85
12			**4** (1 mol%)	6	87
13	4-*^t^*Bu	**5**g	Cu(CH_3_COO)_2_·H_2_O (1 mol%) + **1** (2 mol%)	8	95
14			**4** (1 mol%)	6	97
15	4-NH_2_	**5**h	Cu(CH_3_COO)_2_·H_2_O (1 mol%) + **1** (2 mol%)	8	81
16			**4** (1 mol%)	6	86

^a^ Reaction conditions: alkyne (1 mmol), benzyl azide (1 mmol), 3 mL of water and acetonitrile mixture (1:1), 80 °C.

## Supplementary Materials

The following are available online. Table S1: Crystallographic data and structure refinement details for 2 and 3, Figure S1: 1H NMR spectrum of DAPTA=O (1) in DMSO-*d*6 (500 MHz), Figure S2: 31P{1H} NMR spectrum of DAPTA=O (1) in DMSO-*d*6 (500 MHz), Figure S3: 31P{1H} NMR spectrum of DAPTA=O (1) in D2O (400 MHz), Figure S4: 1H NMR spectrum of DAPTA=S (2) in DMSO-*d*6 (500 MHz), Figure S5: 31P{1H} NMR spectrum of DAPTA=S (2) in DMSO-*d*6 (500 MHz), Figure S6: 31P{1H} NMR spectrum of DAPTA=S (2) in D2O (400 MHz), Figure S7: 1H NMR spectrum of DAPTA=Se (3) in DMSO-*d*6 (500 MHz), Figure S8: 31P NMR spectrum of DAPTA=Se (3) in CDCl3 (400 MHz), Figure S9: 31P{1H} NMR spectra of DAPTA and compounds 1–3 in DMSO-*d*6, Figure S10: Hirshfeld surfaces (top), and shape-index representations of the O⋅⋅⋅H contacts (bottom) of DAPTA and the P-functionalized derivatives 1–3, Characterization data of triazoles (**5**).
