# Supplementary material for: Water-Soluble O-, S- and Se-Functionalized Cyclic Acetyl-triaza-phosphines. Synthesis, Characterization and Application in Catalytic Azide-alkyne Cycloaddition
Source: Molecules. 2020 Nov 23;25(22):5479. doi: 10.3390/molecules25225479 (PMC7700463; doi:10.3390/molecules25225479)
Supplement: Supplementary file 1 [file molecules-25-05479-s001.pdf]

Electronic supplementary information

Water-soluble O-, S- and Se-functionalized cyclic acetyl-triaza-phosphines. Synthesis, characterization and application in catalytic azide-alkyne cycloaddition

Abdallah G. Mahmoud,[1,2]* Piotr Smoleński,[3] M. Fátima C. Guedes da Silva,[2] Armando J. L. Pombeiro[2]

[1] *Department of Chemistry, Faculty of Science, Helwan University, Ain Helwan, 11795 Cairo, Egypt*

[2] *Centro de Química Estrutural, Instituto Superior Técnico, Universidade de Lisboa Av. Rovisco Pais, 1049-001 Lisboa, Portugal*

[3] *Faculty of Chemistry, University of Wrocław, Ul. F. Joliot*-Curie *14, 50-383 Wrocław, Poland.*

* abdallah.mahmoud@tecnico.ulisboa.pt

1. X-ray data

Table S1. Crystallographic data and structure refinement details for **2** and **3**.

	**2**	**3**
Empirical formula	$C_9H_{16}N_3O_2PS$	$C_9H_{16}N_3O_2PSe$
Formula Weight	261.28	308.18
Crystal system	orthorhombic	monoclinic
Space group	Pbcn	P21/n
Temperature/K	298(2)	296(2)
a/Å	25.0532(13)	7.2182(11)
b/Å	8.3441(5)	24.926(3)
c/Å	11.8100(6)	7.5886(11)
α/°	90	90
β/°	90	114.010(7)
γ/°	90	90
V (Å^3)	2468.8(2)	1247.2(3)
Z	8	4
D_{calc} (g cm^{-3})	1.406	1.641
F000	1104	624
μ(Mo Kα) (mm^{-1})	0.382	3.129
Rfls. collected/unique/observed	13312 / 2238 / 1897	2542 / 2542 / 2014
Final $R1^a$, $wR2^b$ ($I \geq 2\sigma$)	0.0451, 0.1062	0.0545, 0.1523
Goodness-of-fit on F^2	1.090	1.059

^{a} R = $\Sigma||F_o|–|F_c||/\Sigma|F_o|$; ^{b} wR($F^2$) = $[\Sigma w(|F_o|^2 – |F_c|^2)^2/\Sigma w|F_o|^4]^{½}$.

2. NMR spectra

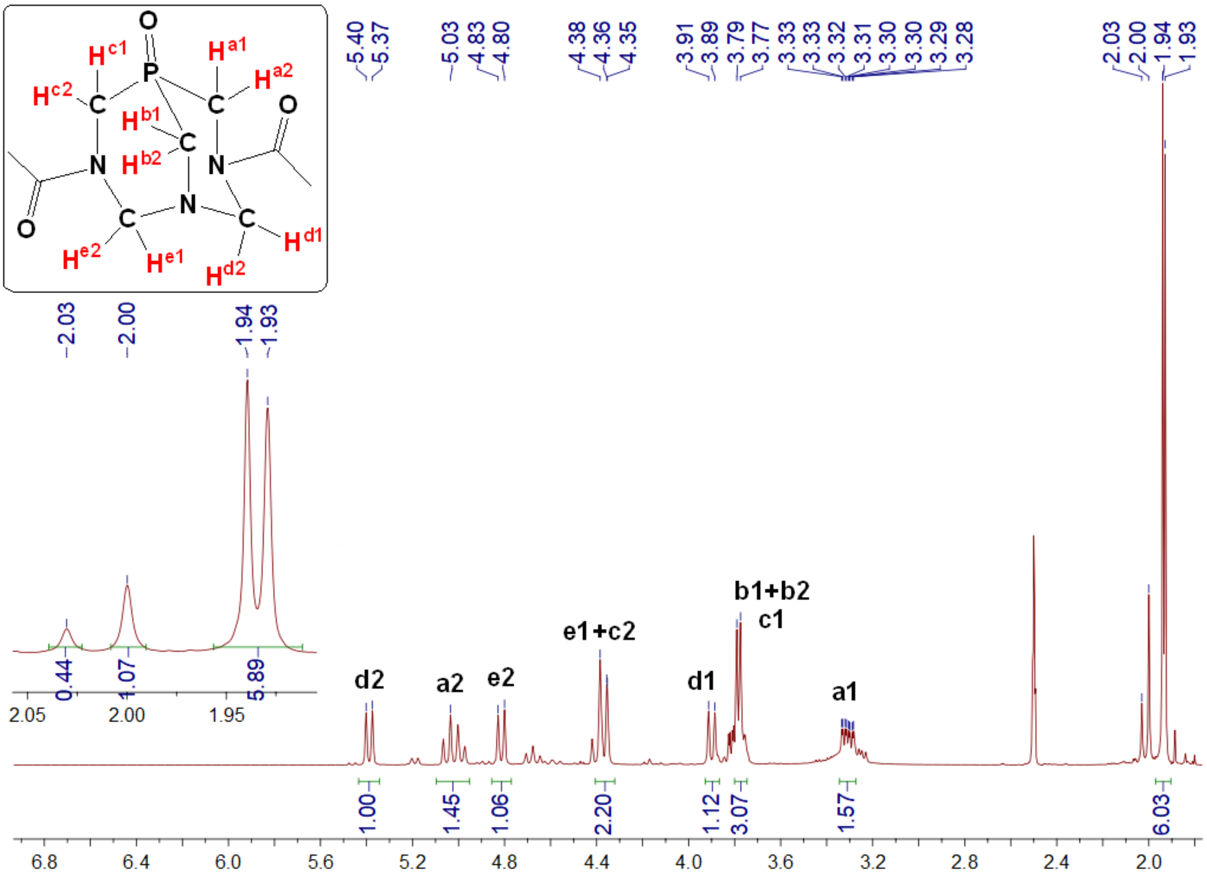

Figure S1. ^{1}H NMR spectrum of DAPTA=O (**1**) in DMSO-d_6 (500 MHz).

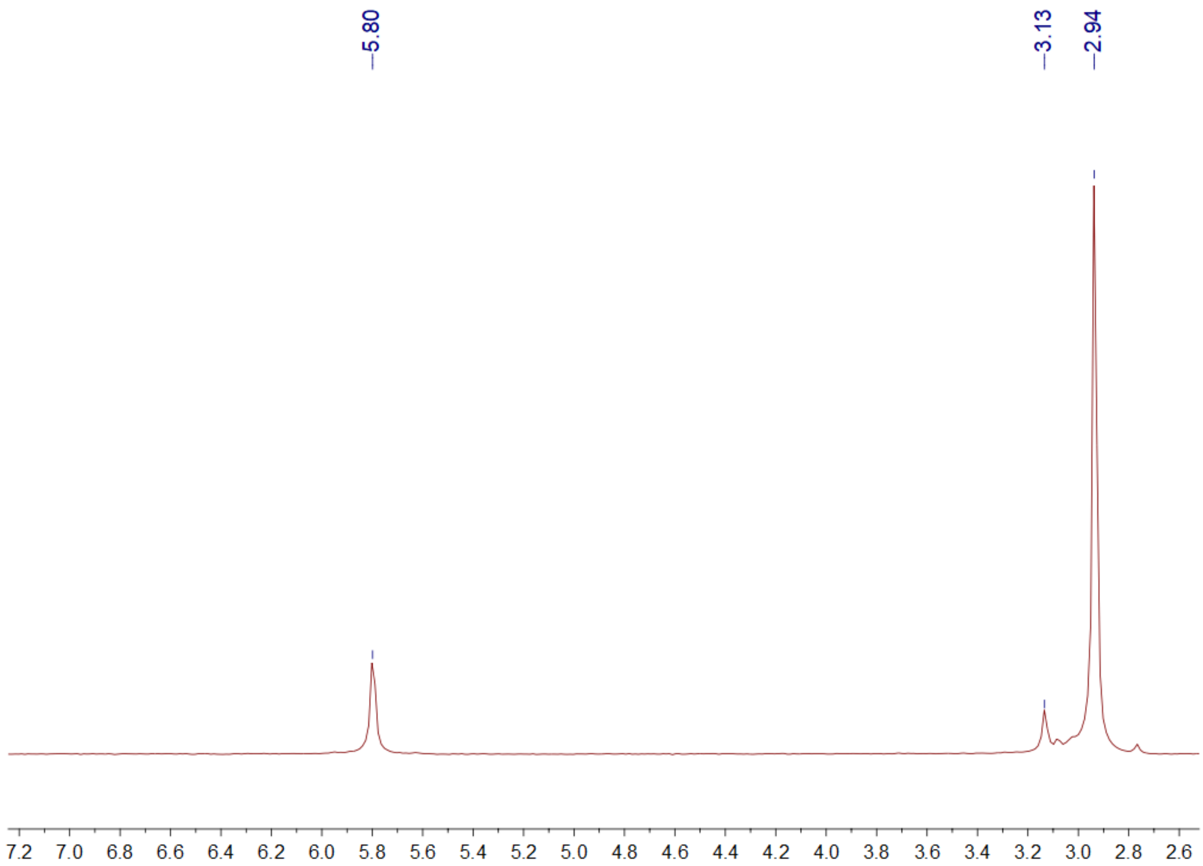

Figure S2. $^{31}P\{^{1}H\}$ NMR spectrum of DAPTA=O (**1**) in DMSO-d_6 (500 MHz).

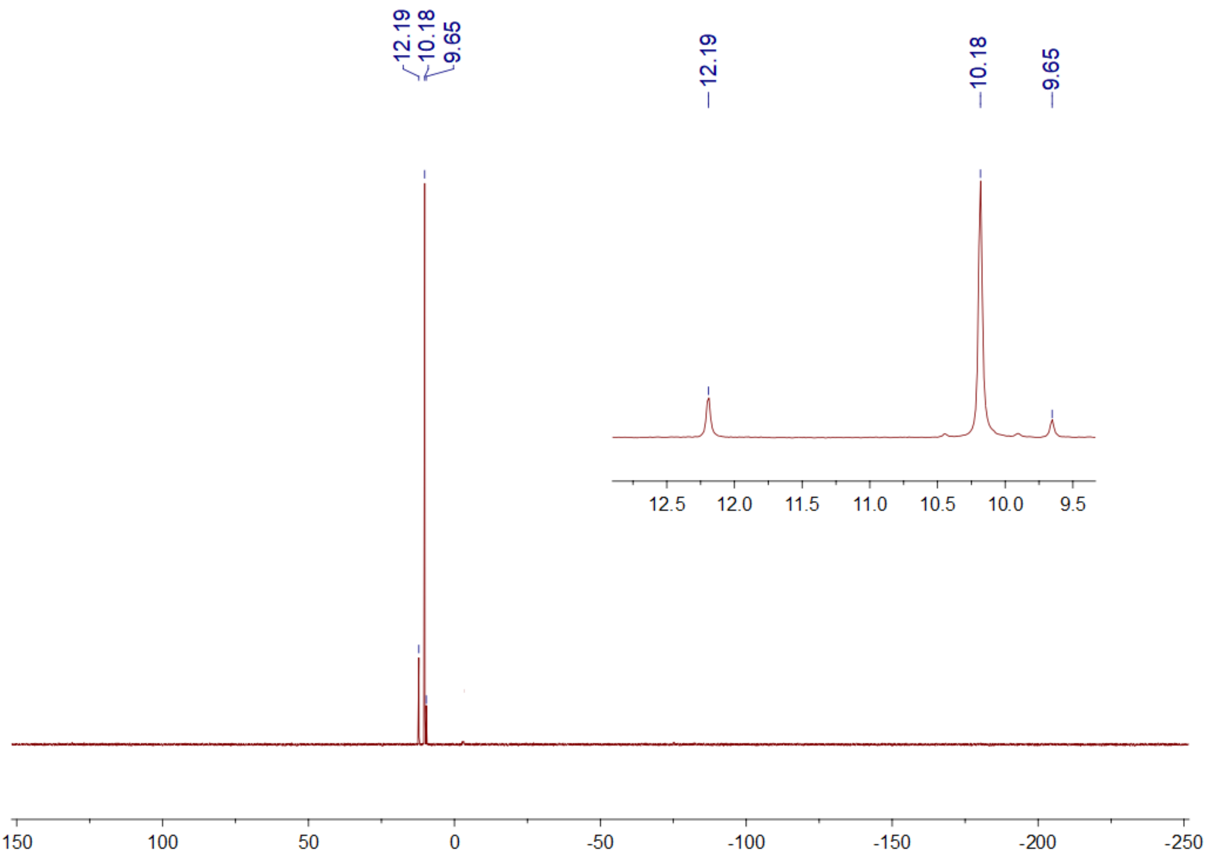

Figure S3. $^{31}P\{^{1}H\}$ NMR spectrum of DAPTA=O (**1**) in D_2O (400 MHz).

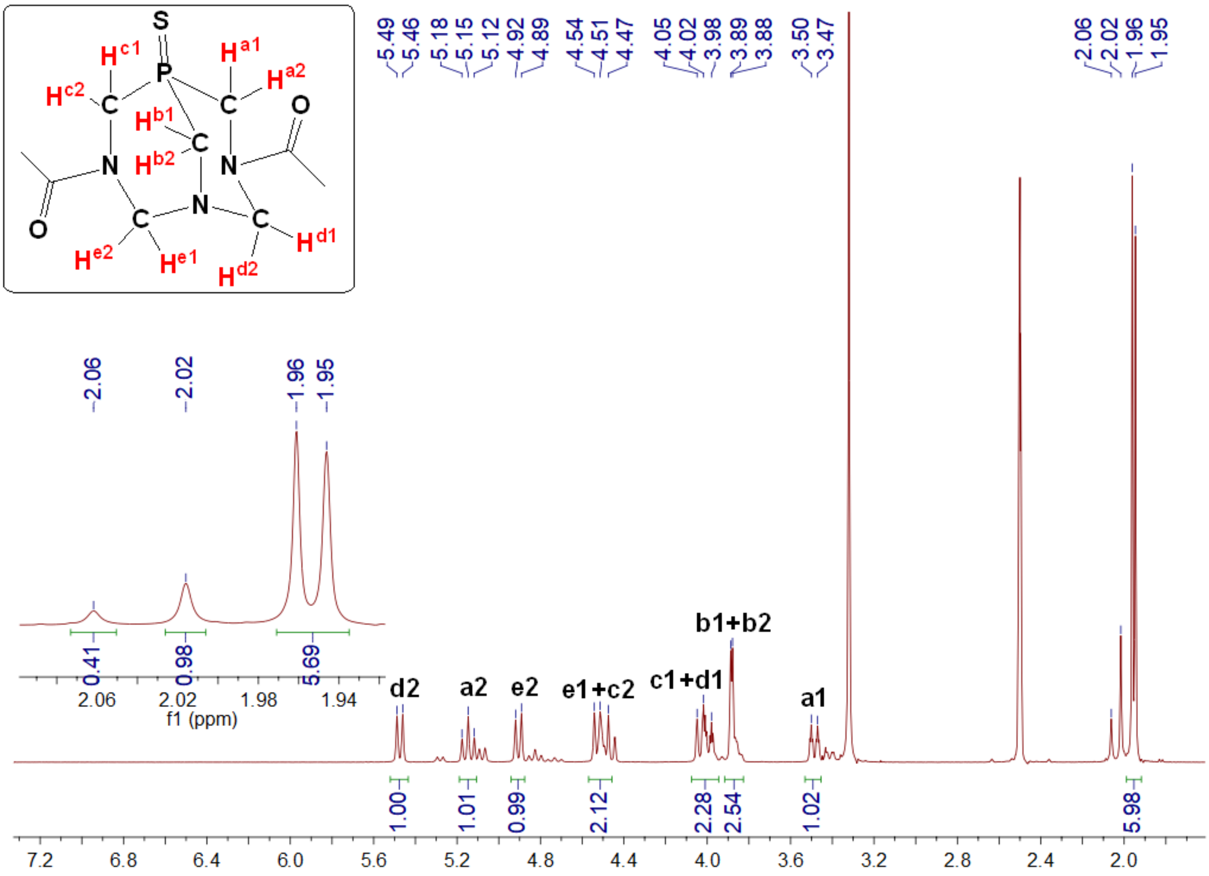

Figure S4. ^{1}H NMR spectrum of DAPTA=S (**2**) in DMSO-d_6 (500 MHz).

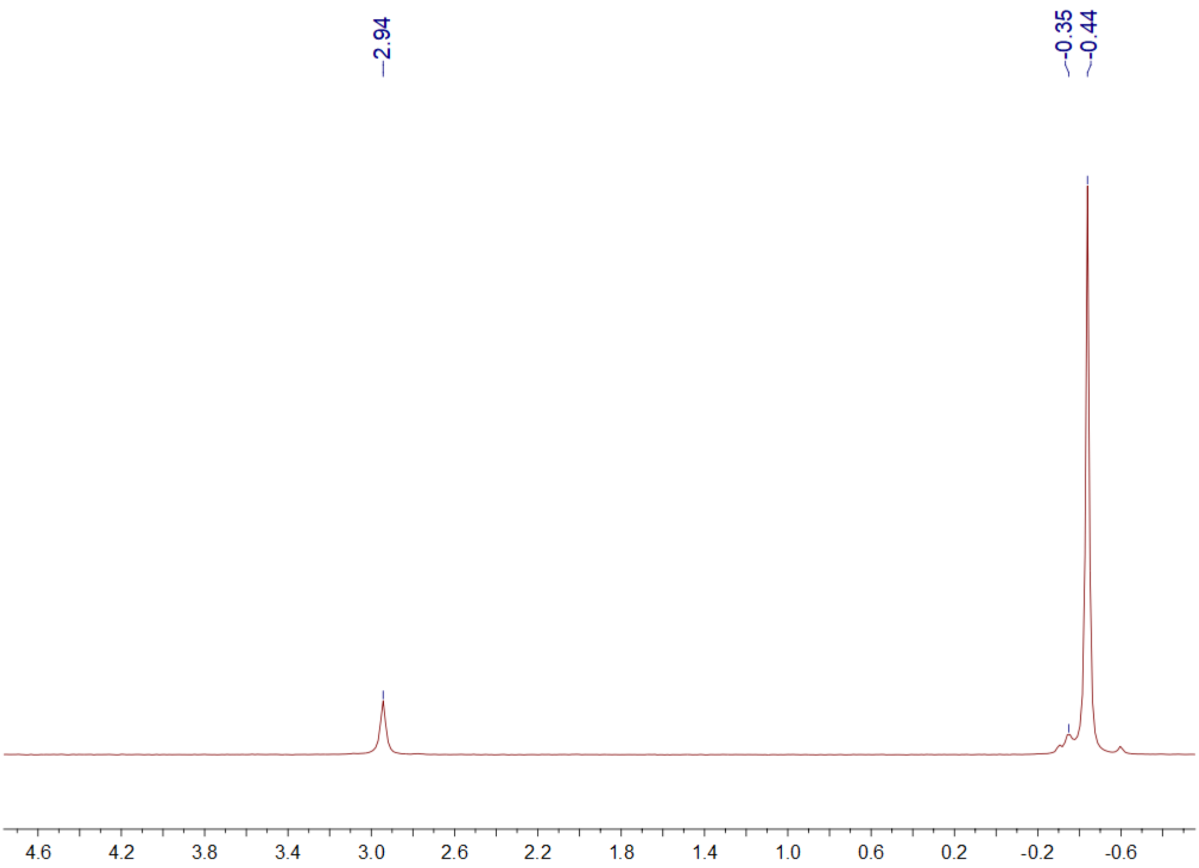

Figure S5. $^{31}P\{^{1}H\}$ NMR spectrum of DAPTA=S (**2**) in DMSO-d_6 (500 MHz).

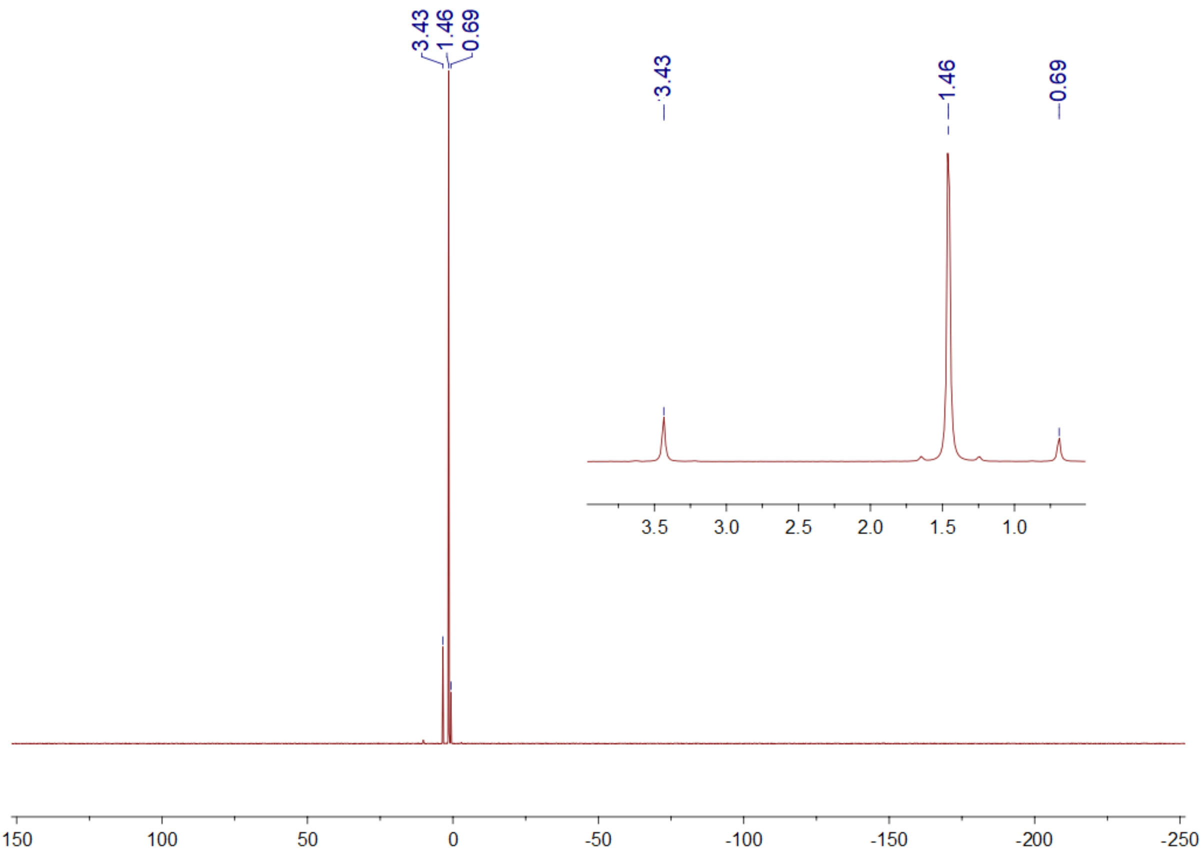

Figure S6. $^{31}P\{^1H\}$ NMR spectrum of DAPTA=S (**2**) in D_2O (400 MHz).

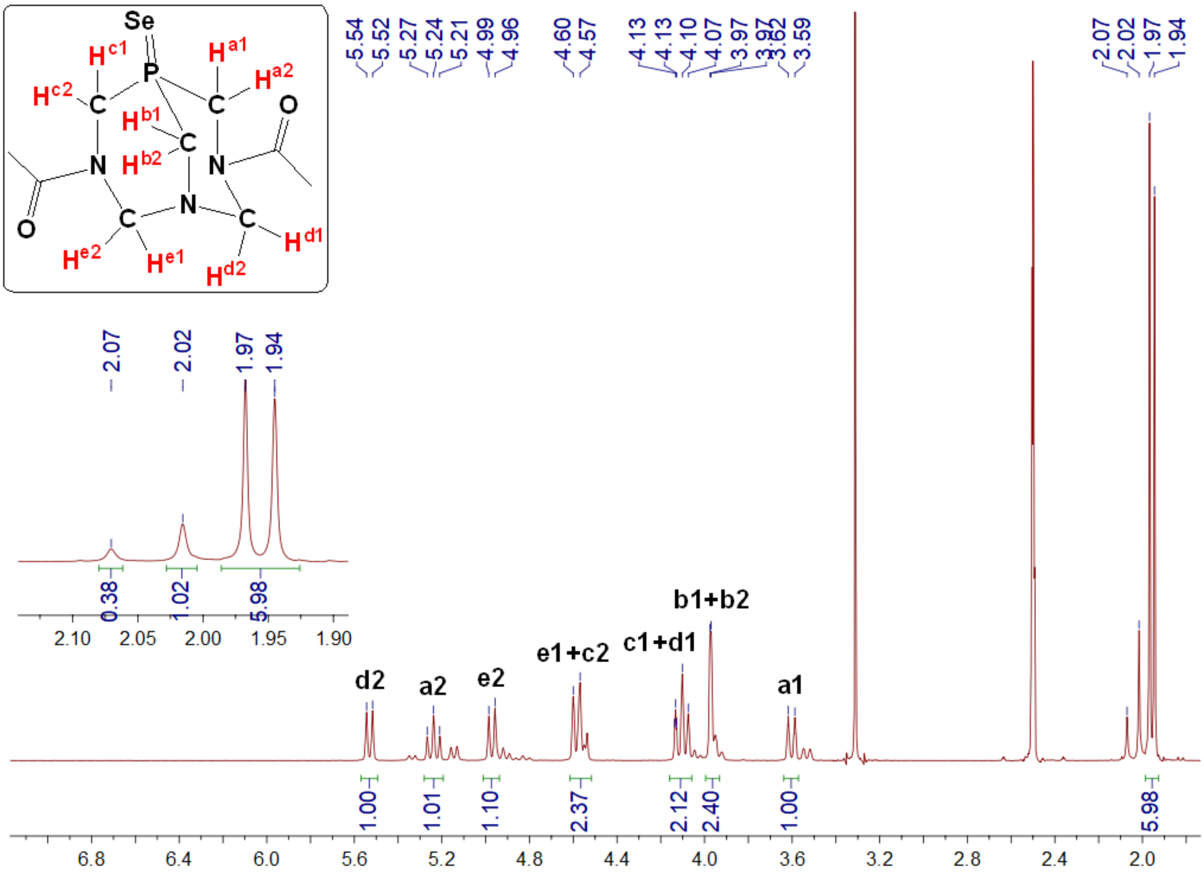

Figure S7. ^{1}H NMR spectrum of DAPTA=Se (**3**) in DMSO-d_6 (500 MHz).

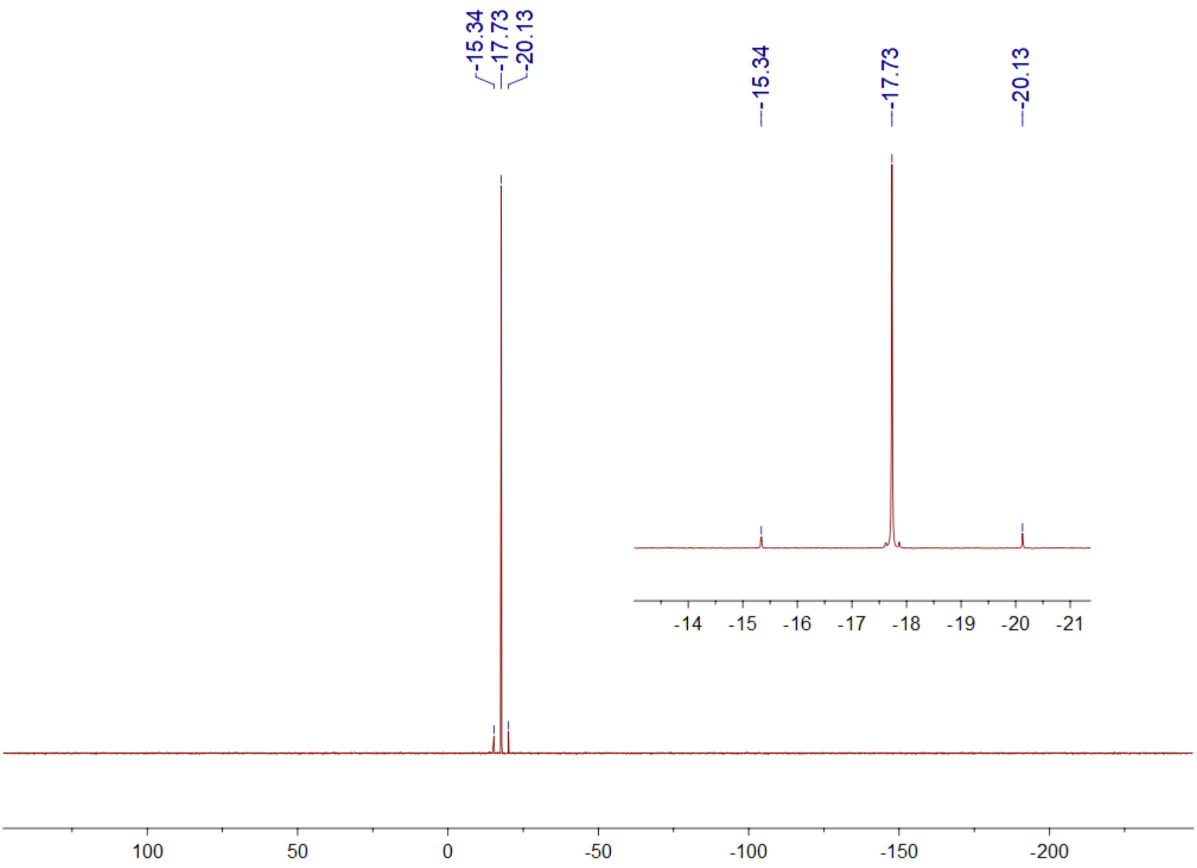

Figure S8. ^{31}P NMR spectrum of DAPTA=Se (**3**) in $CDCl_3$ (400 MHz).

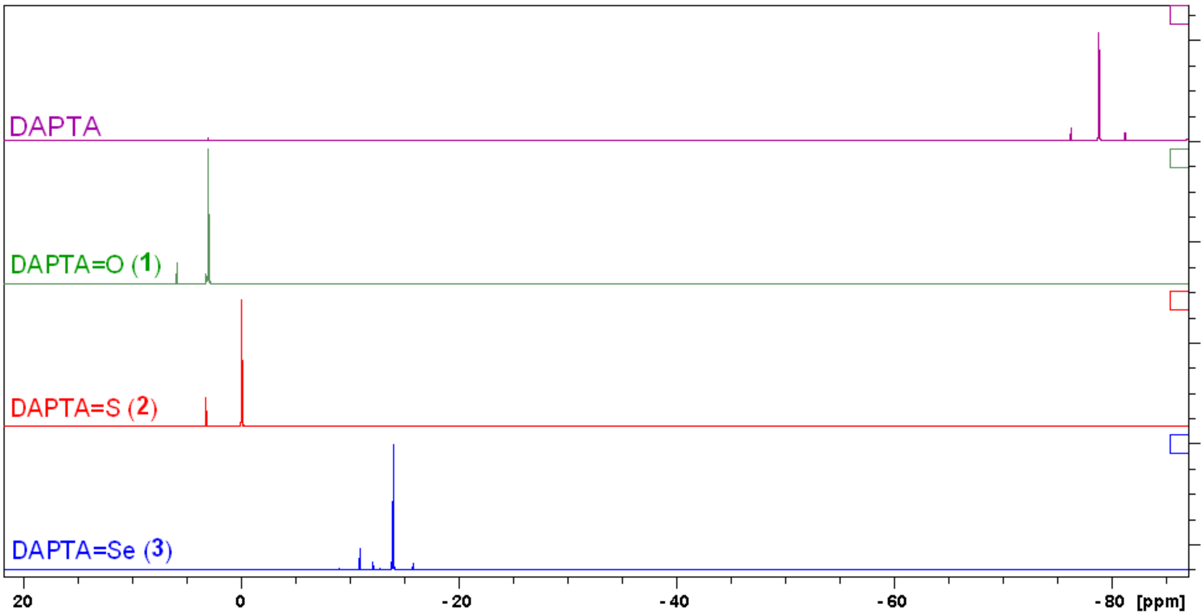

Figure S9. $^{31}P\{^{1}H\}$ NMR spectra of DAPTA and compounds **1-3** in DMSO-d_6.

3. Hirshfeld surfaces analysis

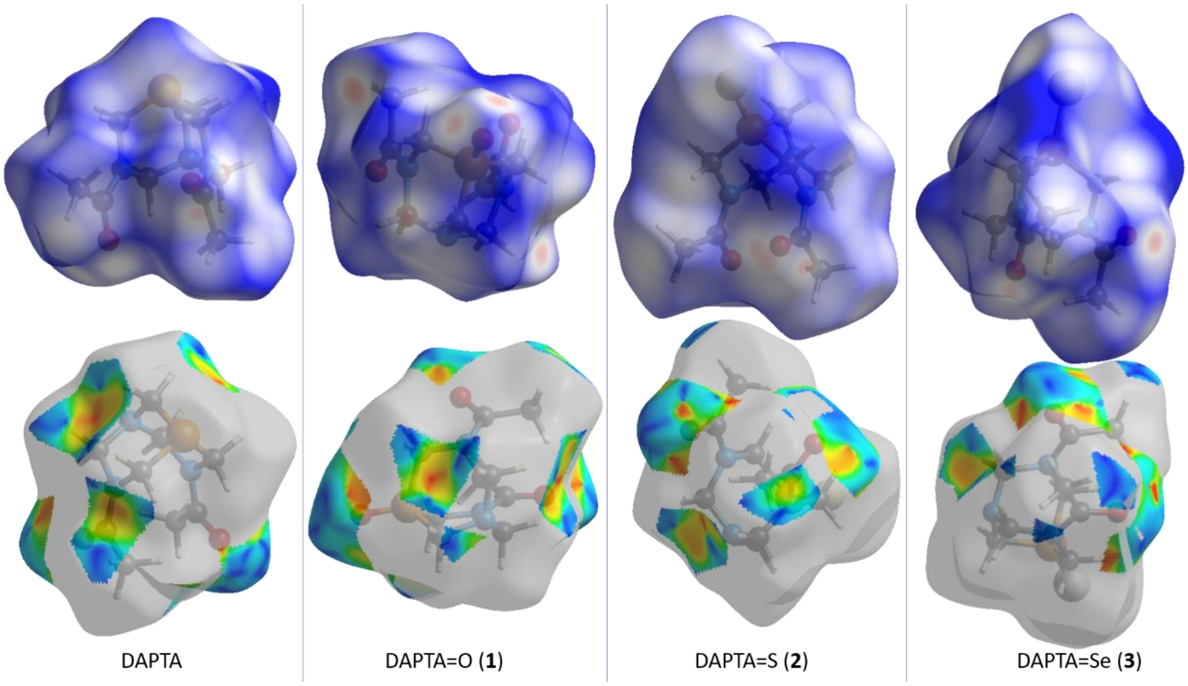

Figure S10. Hirshfeld surfaces (top), and shape-index representations of the O···H contacts (bottom) of DAPTA and the P-functionalized derivatives **1-3**.

4. Characterization data of triazoles (5)

1-benzyl-4-phenyl-1*H*-1,2,3-triazole (**5a**): Elemental analysis calcd (%) for C15H13N3: C 76.57, H 5.57, N 17.86; found: C 76.77, H 5.49, N 17.92. 1H NMR (300 MHz, DMSO-d6, δ): 8.63 (s, 1H), 7.85 (d, J = 7.6 Hz, 2H), 7.45-7.32 (m, 8H), 5.65 (s, 2H).

1-benzyl-4-(m-tolyl)-1*H*-1,2,3-triazole (**5b**): Elemental analysis calcd (%) for $C_{16}H_{15}N_3$: C 77.08, H 6.06, N 16.85; found: C 76.91, H 6.01, N 16.67. ^{1}H NMR (300 MHz, $CDCl_3$, *δ*): 7.61 (br s, 2H, Ar-H), 7.52–7.49 (m, 1H, Ar-H), 7.43–7.27 (m, 6H, Ar-H), 7.14 (m, 1H, Ar-H), 5.49 (s, 2H, PhCH_2N), 2.34 (s, 3H, CH_3).

1-benzyl-4-(3-methoxyphenyl)-1*H*-1,2,3-triazole (**5c**): Elemental analysis calcd (%) for $C_{16}H_{15}N_3O$: C 72.43, H 5.70, N 15.84; found: C 72.25, H 5.64, N 15.72. ^{1}H NMR (300 MHz, $CDCl_3$, *δ*): 7.60 (s, 1H, Ar-H), 7.41–7.22 (m, 8H, Ar-H), 7.74 (m, 1H, Ar-H), 5.48 (s, 2H, PhCH_2N), 3.81 (s, 3H, CH_3).

1-benzyl-4-(p-tolyl)-1*H*-1,2,3-triazole (**5d**): Elemental analysis calcd (%) for $C_{16}H_{15}N_3$: C 77.08, H 6.06, N 16.85; found: C 77.13, H 6.11, N 16.77. ^{1}H NMR (300 MHz, $CDCl_3$, *δ*): 7.63 (d, *J* = 7.9, 2H, Ar-H), 7.54 (s, 1H, Ar-H), 7.30–7.15 (m, 7H, Ar-H), 5.48 (s, 2H, PhCH_2N), 2.31 (s, 3H, CH_3).

1-benzyl-4-(4-ethylphenyl)-1*H*-1,2,3-triazole (**5e**): Elemental analysis calcd (%) for $C_{17}H_{17}N_3$: C 77.54, H 6.51, N 15.96; found: C 77.35, H 6.42, N 16.05. ^{1}H NMR (300 MHz, $CDCl_3$, *δ*): 7.68 (m, 2H, Ar-H), 7.61 (s, 1H, Ar-H), 7.40–7.34 (m, 3H, Ar-H), 7.31–7.25 (m, 4H, Ar-H), 5.49 (s, 2H, PhCH_2N), 2.61 (q, *J* = 7.9 Hz, 2H, CH_2CH$_3$), 1.28 (t, *J* = 7.9 Hz, 3H, CH$_2$CH_3)

1-benzyl-4-(4-fluorophenyl)-1*H*-1,2,3-triazole (**5f**): Elemental analysis calcd (%) for $C_{15}H_{12}FN_3$: C 71.13, H 4.78, N 16.59; found: C 70.98, H 4.66, N 16.43. ^{1}H NMR (300 MHz, $CDCl_3$, *δ*): 7.78 – 7.69 (m, 2H, Ar-H), 7.61 (s, 1H, Ar-H), 7.39–7.22 (m, 5H, Ar-H), 7.16–7.07 (m, 2H, Ar-H), 5.43 (s, 2H, PhCH_2N).

1-benzyl-4-(4-(tert-butyl)phenyl)-1*H*-1,2,3-triazole (**5g**): Elemental analysis calcd (%) for $C_{19}H_{21}N_3$: C 78.32, H 7.26, N 14.42; found: C 78.25, H 7.22, N 14.37. ^{1}H NMR (300 MHz, $CDCl_3$, *δ*): 7.71 (d, *J* = 7.9 Hz, 2H, Ar-H), 7.63 (m, 1H, Ar-H), 7.40 (d, *J* = 7.9 Hz, 2H, Ar-H), 7.34–7.28 (m, 4H, Ar-H), 5.52 (s, 2H, PhCH_2N), 1.35 (s, 9H, CH_3).

4-(1-benzyl-1*H*-1,2,3-triazol-4-yl)aniline (**5h**): Elemental analysis calcd (%) for $C_{15}H_{14}N_4$: C 71.98, H 5.64, N 22.38; found: C 72.09, H 5.57, N 22.52. ^{1}H NMR (300 MHz, $CDCl_3$, *δ*): 7.63–7.55 (m, 3H, Ar-H), 7.39–7.25 (m, 5H, Ar-H), 6.81–6.74 (m, 2H, Ar-H), 5.58 (s, 2H, PhCH_2N), 3.67 (br s, 2H, NH_2).
